# Acute Myeloid Leukemia in a Patient With Human Immunodeficiency Virus-Related High-Risk Myelodysplastic Syndrome

**DOI:** 10.7759/cureus.32409

**Published:** 2022-12-11

**Authors:** Sneha Bijoy, Sini Bijoy, Sobha Atluri, Folasade Ajayi, Hamid Shaaban

**Affiliations:** 1 Internal Medicine, New York Medical College, Westchester, USA; 2 Hematology/Oncology, St. Michael's Medical Center, Newark, USA; 3 Hematology/Oncology, New York Medical College, Westchester, USA

**Keywords:** aids, human immuno-deficiency virus (hiv)), human immuno deficiency virus, hiv aids, severe pancytopenia, aids ( acquired immunodeficiency syndrome ), myelodysplastic syndrome, acute myeloid leukemia (aml), human immunodeficiency virus infection

## Abstract

The human immunodeficiency virus (HIV) is a retrovirus that infects a subset of T lymphocytes referred to as CD4 T-helper cells. This insult to the quantity and quality of T lymphocytes leads to a significant compromise of the immune system and the development of an environment of abnormal immune activation. This aberrancy in the immune system increases the susceptibility to developing various malignancies. Hematological abnormalities like cytopenias are among the most common complications of HIV and acquired immunodeficiency syndrome (AIDS)-related lymphoid malignancies. Myelodysplastic syndrome (MDS), a disease of ineffective hematopoiesis causing dysplastic cells and hypercellular bone marrow, manifesting as pancytopenia, has been described in patients with HIV but is poorly documented in the medical literature. We present the case of a middle-aged male with longstanding HIV who developed severe pancytopenia secondary to high-risk MDS and eventually progressed to and died from acute myeloid leukemia (AML), a phenomenon infrequently reported as associated with HIV/AIDS. Patients with HIV/AIDS and cytopenias should get a detailed hematological evaluation so as not to miss or delay the AML diagnosis.

## Introduction

Living with the human immunodeficiency virus (HIV) often indicates an increased likelihood of developing malignancies compared to the general population. HIV patients with malignancies typically have a more serious disease process characterized by an earlier age of diagnosis, high tumor grades, rapid disease progression, and poor response to treatments [1]. The various types of malignancies can range from AIDS-defining cancers like Non-Hodgkin’s lymphoma (NHL) and Kaposi sarcoma to those that can be associated with HIV like melanoma, gastrointestinal, genitourinary, breast, and lung cancer [1-2]. Although the increased incidence of certain malignancies, such as NHL and Kaposi sarcoma, in patients with HIV is well established, the risk of developing myelodysplastic syndrome (MDS) is not. MDS is a group of blood disorders characterized by an abnormal maturation of cells, cytopenias, and bone marrow failure [3]. MDS and acute myeloid leukemia (AML) in HIV/AIDS patients are very rare. Herein, we report the case of a 56-year-old male with acquired immunodeficiency syndrome (AIDS), non-compliant with highly active antiretroviral therapy (HAART), who presented with fatigue and intractable epistaxis with laboratory evidence of pancytopenia. The latter prompted a bone marrow biopsy, which revealed MDS. He subsequently developed AML and succumbed to disease complications shortly thereafter.

## Case presentation

A 56-year-old Hispanic male with a past medical history of HIV/AIDS and non-compliance with HAART, hepatitis C status post-treatment with a sustained virologic response, and poly-substance use (tobacco, alcohol, marijuana, and heroin) presented to the emergency department with generalized weakness and a two-week history of spontaneous epistaxis refractory to prolonged manual pressure. Blood work on admission was significant for pancytopenia (Table 1). Markers for hemolysis such as lactate dehydrogenase and haptoglobin were within normal limits. The fibrinogen level was elevated (Table 2), ruling out disseminated intravascular coagulation (DIC). The iron panel was consistent with anemia, a chronic disease. Folate and vitamin B12 levels were adequate (Table 3). The low reticulocyte index was consistent with hypoproliferation of the bone marrow. His labs revealed a high HIV viral load with a low absolute cluster of differentiation 4 (CD4) count (Table 2).

**Table 1:** Lab results of a complete blood cell count with differential

Hemogram	Result	Reference interval
White blood cell count (10*3/uL)	1.60	4.40 – 11.00
Red blood cell count (10*6/uL)	2.13	4.32 – 5.72
Hemoglobin (g/dL)	6.6	13.5 – 17.5
Hematocrit (%)	19.1	38.8 – 50.0
Mean corpuscular volume (fL)	89.7	81.2 – 95.1
Mean corpuscular hemoglobin (pg)	31.1	27.5 – 33.2
Mean corpuscular hemoglobin concentration (g/dL)	34.6	33.4 – 35.5
Red blood cell distribution width (%)	16.5	11.8 – 15.6
Platelets (10*3/uL)	16	150 – 450
Mean platelet volume (fL)	7.1	7.4 – 11.0
Auto differential	Result	Reference interval
Lymphocytes (relative) (%)	48.6	None
Monocytes (relative) (%)	20.0	None
Neutrophils (relative) (%)	30.4	None
Eosinophils (relative) (%)	0.6	None
Basophils (relative) (%)	0.4	None
Neutrophils (absolute) (10*3/uL)	0.5	1.7 – 7.0
Lymphocytes (absolute) (10*3/uL)	0.8	0.9 – 2.9
Monocytes (absolute) (10*3/uL)	0.3	0.3 – 0.9
Eosinophils (absolute) (10*3/uL)	0.0	0.0 – 0.5
Basophils (absolute) (10*3/uL)	0.0	0.0 – 0.2

**Table 2:** Laboratory values RNA: ribonucleic acid; CD3: cluster of differentiation 3; CD4: cluster of differentiation 4; CD8: cluster of differentiation 8

Others	Result	Reference interval
Lactate dehydrogenase (U/L)	141	122 - 222
Haptoglobin (mg/dL)	69.6	30 - 200
Fibrinogen (mg/dL)	544	200 – 393
Reticulocyte count (%)	3.6	0.5 – 1.5
Reticulocyte index (%)	0.6	
HIV-1 RNA by polymerase chain reaction (copies/mL)	174,920	20 – 10,000,000
Absolute CD3 (/uL)	707	622 – 2402
Absolute CD4 helper (/uL)	12	359 – 1519
Absolute CD8 suppressor (/uL)	662	109 – 897
% CD3-positive lymphocytes (%)	88.4	57.5 – 86.2
% CD4-positive lymphocytes (%)	1.5	30.8 – 58.5
% CD8-positive lymphocytes (%)	82.7	12.0 – 35.5
CD4/CD8 ratio	0.02	0.92 – 3.72

**Table 3:** Laboratory results for the iron panel, vitamin B12, and folate

Iron/Anemia profile	Result	Reference interval
Iron (ug/dL)	35	50 – 150
Ferritin (ng/mL)	2,100.3	24.0 – 336.0
Iron saturation (%)	23.8	20.0 – 55.0 %
Total iron-binding capacity (ug/dL)	147.0	250.0 – 400.0
Folate (ng/ml)	19.0	5.4 – 25
Vitamin B12 (pg/mL)	1,346.0	180.0 – 914.0

**Figure 1:**
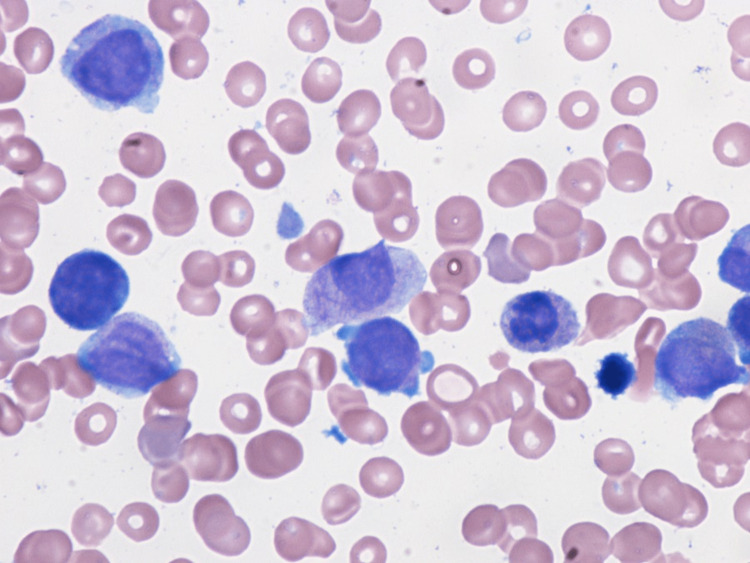
Myeloblasts seen in a peripheral blood smear (yellow arrow)

**Figure 2:**
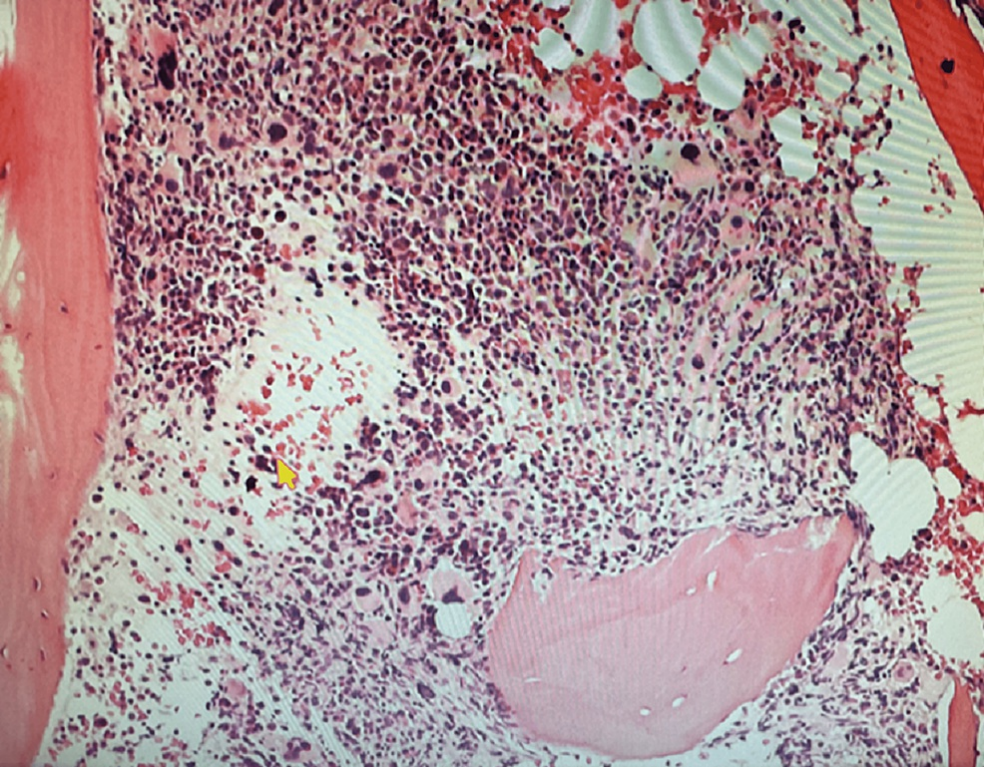
A bone marrow core biopsy revealed 100% hypercellular marrow

**Figure 3:**
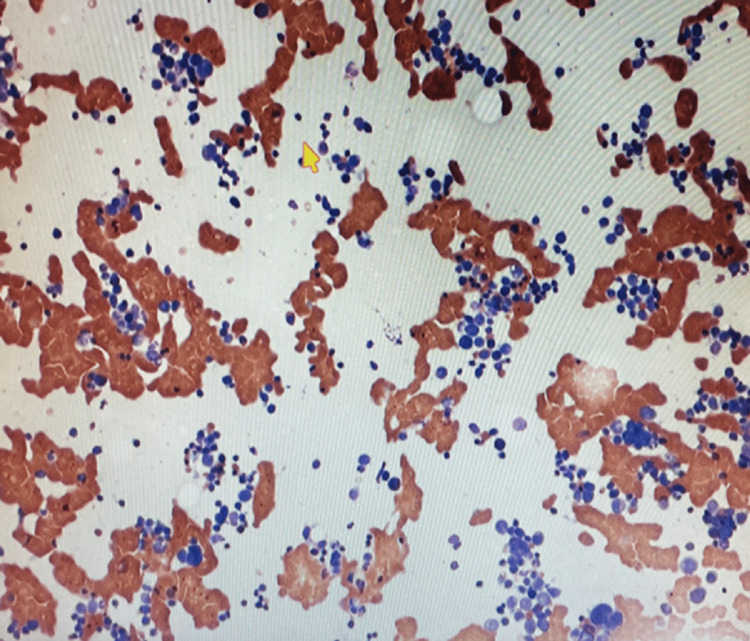
Myeloblast (yellow arrow) seen on a bone marrow smear

**Figure 4:**
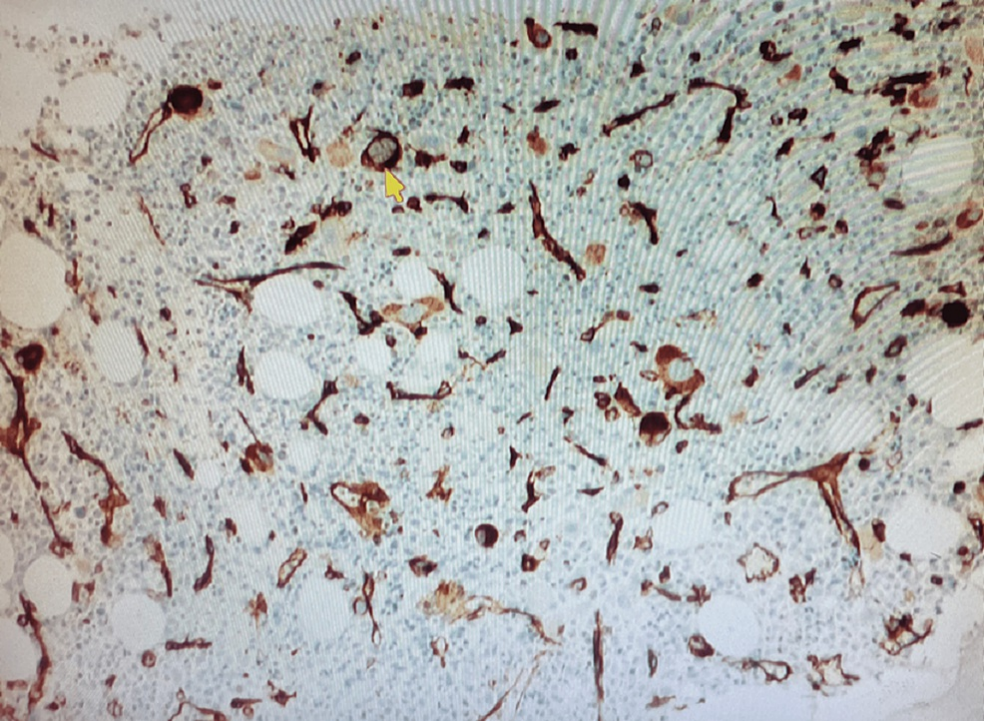
Immunohistochemical staining of a bone marrow core biopsy for CD34, a marker for myeloblasts, shows increased blasts (yellow arrow)

A peripheral blood smear showed pancytopenia and thrombocytopenia with scattered dysmorphic hypo-segmented neutrophils, rare nucleated red blood cells, and rare giant platelets. Myeloblasts (Figure 1) were also seen on the peripheral smear. Following a bone marrow biopsy, myelodysplastic syndrome with excess blasts 1 (MDS-EB1) was discovered in a markedly hypercellular marrow (>80%) for age, with left-shifted maturation and trilineage dysplasia (Figure 2). Blasts (Figure 3) were increased by 8% by differential count and immunohistochemistry (Figure 4). Mild reticulin fibrosis (MF-1) was also noted. A fluorescence in situ hybridization (FISH) study detected MDS-related abnormalities, including monosomy 7 and 5q deletion. Karyotyping study showed 43,XY, del(5)(q22q35), -7, dic(9;15)(q12;p12), der(12)t(12;21)(p11.2)(6)/44, sl, +idic(9)(q12)(8)/43~44, sdl, -dic(9;15), +0~1mar (cp5)/46,XY(1). A molecular study with the Illumina paired-end sequencing Next Generation Sequencing platform revealed the TP53 mutation (60%). The complex karyotype and TP53 mutation portend an unfavorable prognosis in MDS. The age-adjusted Revised International Prognostic Scoring System (IPSS-RA) score was 8.9, consistent with a very high risk for MDS.

During his hospitalization, the patient received five units of packed red blood cells (pRBCs). Since the platelet count was <20,000/uL with active bleeding, the patient was also transfused a total of two units of platelets. Additionally, he was given two doses of intravenous immunoglobulin (IVIG) to address HIV-related immune thrombocytopenia (ITP) with a platelet count of 15K/uL. He was also given granulocyte colony-stimulating factors to increase WBC and erythropoietin analogs to treat HIV-related anemia. Folic acid and a multivitamin were added for additional hematopoietic support. Blood counts stabilized, and epistaxis eventually resolved with nasal packing and Afrin nasal spray. The patient was counseled on the cessation of alcohol use, which results in myelosuppression, and educated on the importance of compliance with HAART to increase CD4 counts. The patient was subsequently discharged to subacute rehab.

Approximately one month later, the patient was admitted to a cancer institute for the treatment of MDS. He received five days of the hypomethylating agent, decitabine, and continued to receive blood product support as needed for severe pancytopenia. He was not deemed a candidate for an allogeneic stem cell transplant due to his poor performance status, non-compliance with medical follow-up and treatment, and psychosocial issues. Over the next few months, the patient had multiple admissions to other hospitals for epistaxis and severe pancytopenia. Increased blasts were discovered in the peripheral blood smear, and it was concluded that the patient had progressed to AML. The patient ultimately succumbed to his disease shortly after diagnosis.

## Discussion

The occurrence of MDS is poorly studied, and the incidence of MDS in patients living with HIV is even more so. About 20,000 new cases of MDS are reported each year in the US. Approximately 60,000-170,000 patients in the US are living with MDS, with the propensity to progress into AML seen in 30% of cases [4-6]. It is well known that HIV heralds a poor prognosis for many malignancies. However, the disease course of MDS/AML in the setting of HIV infection is poorly documented in the literature, barring a handful of case reports. In the cohort study by Kaner et al., the incidence and clinical manifestations of proven MDS or AML in HIV-positive patients were compared to those of MDS or AML diagnosed in HIV-negative patients. The study confirmed that there was indeed a suppression of hematopoiesis specific to HIV infection [5]. The study brought awareness to the prevalence of MDS in the HIV population and the need for early diagnosis and implementation of treatment in this already hapless population [5].

Hematological abnormalities are commonly seen in patients with HIV. Laboratory evaluations show cytopenias of varying degrees, including leukopenia, anemia, and thrombocytopenia, often occurring concomitantly. Although many theories exist for the possible etiology and explanation of these findings, it is worth mentioning that the answer is likely multifactorial. Treatment of HIV infection necessitates multi-drug regimens that are often cytotoxic, superimposed by opportunistic infections that suppress bone marrow production, all collectively blurring the lines between disease and medication or acquired infection-induced cytopenias [6-10].

Myelodysplasia is common with HIV infection, but progression to AML is not. In a retrospective study conducted in France in 2001, data on patients who were diagnosed with AML in the setting of HIV infection were collected from the years 1990 through 1996 [7]. The aim of the study was to compare the occurrence of AML in patients with HIV to that of the general population of France. It was concluded that the incidence of AML in patients with HIV was twice as great when compared to the general population of France [7]. This scarcely documented increase in the development of AML in patients with HIV is hypothesized to be multifactorial. Moses et al. reported that the failure of bone marrow seen in this patient population was likely due to acquiring the infection and disrupting the regulatory roles of various cells of the immune response [8]. Because of the paucity of studies and literature on AML in patients with HIV, it is unclear whether HIV aids in the transformation of progenitor cells or if the compromised immune system fails to regulate transformed cells. Ineffective hematopoiesis can result from the direct suppression of progenitor cells by HIV infection. It can also occur indirectly through continuous immune stimulation. Opportunistic infections, neoplasms, nutritional deficiencies, and medication side effects can all contribute to ineffective hematopoiesis in patients with HIV [9-11]. As reported by Forghieri et al., a proposed theory of HIV increasing the risk of developing MDS or AML is the disruption of the bone marrow microenvironment by the infection [4]. This in turn creates an inflammatory atmosphere and subsequent activation of cytokines, especially those involved in the production and maturation of leukemic stem cells [4]. Another theory, again reported by Forghieri et al., is attributed to the release of the trans-activator protein (Tat) during acute infection of CD4+ T cells. This protein is able to displace the basic fibroblast growth factor (bFGF), which is known to increase myelopoiesis. Additionally, as compared to the general population, prior radiation or chemotherapy exposure is also documented to increase the risk of developing MDS or AML in patients with HIV [10-11].

The treatment for HIV-positive patients with AML is typically the standard AML induction and consolidation chemotherapy. The percentage of patients who achieve complete remission (CR) with this regimen is 73%-83% [7, 9]. However, a high relapse rate is still observed in AML patients who previously achieved CR [9]. Karyotype and CD4 cell count are considered to be predictors of overall survival for HIV-associated AML. Evans et al. studied 31 patients with HIV-related AML and reported that the median survival for favorable and intermediate-risk karyotype patients with CD4 cell counts less than 200 cells/mm3 was 8.5 months compared to 48 months for those with CD4 cell counts greater than 200 cells/mm3 [12]. Our patient had a very low CD4 count and also had a very unfavorable karyotype with very complex cytogenetics, including a tumor protein 53 (TP53) mutation, which typically portends a poor prognosis. Another possible treatment option is allogenic (allo) hematopoietic stem cell transplantation (HSCT). Hagiwara et al. reported that two patients with HIV-related AML survived for more than four years after receiving high-dose chemotherapy, followed by allo-HSCT [1].

In 2009, Hütter et al. reported the case of an AML patient who achieved complete remission with HIV eradication and sustained remission without ART following allo-HSCT. The patient received stem cells from a donor who was homozygous for CCR5-D32, a nonfunctional allele of the CCR5 coreceptor used by HIV to infect human cells [13].

## Conclusions

Although people living with HIV/AIDS have an increased predilection for developing hematological malignancies, the risk of MDS and progression to AML is scarcely reported. This paucity of data is likely due to the complex disease process of HIV and the multifaceted etiology of ineffective hematopoiesis seen in this population. There are multiple theories, as stated above, for the development of AML in these patients, such as the role of ineffective T cell regulation on hematopoiesis and defective immune surveillance. This rare case report of AIDS-related MDS with progression to AML highlights the importance of compliance with HAART to increase CD4 count, which ultimately is the mainstay of treatment of HIV-related myelosuppression and MDS. This patient’s complex karyotype and TP53 mutation make it an exceedingly rare case of high-risk MDS with subsequent progression to AML.
